# A Cone-Shaped Phantom for Assessment of Small Animal PET Scatter Fraction and Count Rate Performance

**DOI:** 10.1007/s11307-012-0546-2

**Published:** 2012-02-07

**Authors:** Rameshwar Prasad, Habib Zaidi

**Affiliations:** 1 Division of Nuclear Medicine and Molecular Imaging, Geneva University Hospital, 1211 Geneva, Switzerland; 2 Geneva Neuroscience Center, Geneva University, 1211 Geneva, Switzerland; 3 Department of Nuclear Medicine and Molecular Imaging, University Medical Center Groningen, University of Groningen, 9700 Groningen, the Netherlands

**Keywords:** PET, Small animals, Scatter, Count rate, Monte Carlo simulation

## Abstract

**Purpose:**

Positron emission tomography (PET) image quality deteriorates as the object size increases owing to increased detection of scattered and random events. The characterization of the scatter component in small animal PET imaging has received little attention owing to the small scatter fraction (SF) when imaging rodents. The purpose of this study is first to design and fabricate a cone-shaped phantom which can be used for measurement of object size-dependent SF and noise equivalent count rates (NECR), and second, to assess these parameters for two small animal PET scanners as function of radial offset, object size and lower energy threshold (LET).

**Methods:**

The X-PET™ and LabPET-8™ scanners were modeled as realistically as possible using GATE Monte Carlo simulation platform. The simulation models were validated against experimental measurements in terms of sensitivity, SF and NECR. The dedicated phantom was fabricated in-house using high-density polyethylene. The optimized dimensions of the cone-shaped phantom are 158 mm (length), 20 mm (minimum diameter), 70 mm (maximum diameter) and taper angle of 9°.

**Results:**

The relative difference between simulated and experimental results for the LabPET-8™ scanner varied between 0.7% and 10% except for a few results where it was below 16%. Depending on the radial offset from the center of the central axial field-of-view (3–6 cm diameter), the SF for the cone-shaped phantom varied from 26.3% to 18.2%, 18.6 to 13.1% and 10.1 to 7.6% for the X-PET™, whereas it varied from 34.4% to 26.9%, 19.1 to 17.0% and 9.1 to 7.3% for the LabPET-8™, for LETs of 250, 350 and 425 keV, respectively. The SF increases as the radial offset decreases, LET decreases and object size increases. The SF is higher for the LabPET-8™ compared with the X-PET™ scanner. The NECR increases as the radial offset increases and object size decreases. The maximum NECR was obtained at a LET of 350 keV for the LabPET-8™ and 250 keV for the X-PET™. High correlation coefficients for SF and NECR were observed between the cone-shaped phantom and an equivalent volume cylindrical phantom for the three considered axial fields of view.

**Conclusions:**

A single cone-shaped phantom enables the assessment of the impact of three factors, namely radial offset, LET and object size on PET SF and count rate estimates. This phantom is more realistic owing to the non-uniform shape of rodents’ bodies compared to cylindrical uniform phantoms and seems to be well suited for evaluation of object size-dependent SF and NECR.

## Introduction

Positron emission tomography (PET), a non-invasive molecular imaging modality, is being widely used for qualitative characterization and quantification of biochemical processes *in vivo*. PET scanners dedicated to small-animal imaging provide the high spatial resolution and high sensitivity required for *in vivo* molecular imaging [1]. This steered the development of various innovative design concepts and technologies for preclinical PET imaging [2].

The image quality and quantitative accuracy of PET images are degraded by many physical factors such as the attenuation of photons, the detection of scattered photons and the finite spatial resolution of the imaging system. Scattered events decreases image contrast by misplacing events while assigning to a line of response, thus causing bias resulting in the overestimation of the actual activity concentration. The scatter fraction (SF), defined as the ratio of scattered coincidences to total coincidences, is a useful parameter to assess the magnitude of scatter and to estimate its impact on reconstructed PET images. Likewise, the noise equivalent count rate (NECR) is an important parameter used for performance characterization of PET systems [3]. The SF largely depends on object size and density, energy window settings and scanner geometry. The magnitude of SF has been widely studied and is well documented for clinical PET imaging. It represents 10% to 20% in two-dimensional (2D) mode, whereas it reaches 30–35% in brain imaging and 50–60% in whole-body imaging in three-dimensional (3D) acquisition mode [4]. Though the characterization of the scatter component in small animal PET imaging has received little attention owing to the small SF, SF estimates have been reported for various small animal PET scanners [5]. Depending on PET system characteristics and acquisition parameters, the typical range is 5% to 21% for mice and 15% to 30% for rats [6–13]. The SF and NECR are usually measured using various discrete phantoms of different uniform size [14–16]. For instance, the National Electrical Manufacturers Association (NEMA) standards [17] suggest three different phantoms for SF and count rate measurements for small animal PET scanners. These correspond to mouse-, rat- and monkey-sized phantoms of 25, 50 and 100 mm diameter, respectively. However, within a specific rodent species, especially rats or small rabbits, there is substantial variation in body size when rodents are litters or correspond to diabetic models put on high calorie diets. Also, the rodents’ body shape is not uniform throughout in the axial direction. The body cross-section of rodents’ specially rat and large species increases from head towards pelvis region. Moreover, it has been suggested that a phantom representing a varying range of cross-sections and dimensions would be more suited for the assessment of these parameters for clinical PET systems [18]. Furthermore, it is nowadays common practice to increase the throughput of rodent PET studies by simultaneous scanning of multiple rodents placed at different radial offsets in the scanner’s fields of view (FOV) [19].

The purpose of this study is, first, to design and develop a cone-shaped phantom for the measurement of object size-dependent SF and NECR and, second, to assess these parameters as function of radial offset, object size and lower energy threshold (LET) for two small animal PET scanners, namely the X-PET™ and LabPET-8™ using the developed cone-shaped phantom.

## Materials and Methods

### Modeling and Validation of Small Animal PET Scanners

Both the X-PET™ and LabPET-8™ scanners were modeled as realistically as possible in terms of geometry, physics of photon transport and signal processing using the GATE Monte Carlo simulation toolkit [20]. This package is based on the well-established Geant4 libraries [21] featuring a modular, versatile, scripted simulation toolkit. As such, it allows accurate modeling of detailed scanner geometries and radiation interaction with matter, tracking events (recording position, time and energy of an event) and creating various types of output files including projection data and other relevant parameters. Simulation models of both scanners were validated through comparison to experimental measurements, namely sensitivity, NECR and SF for mouse and rat-sized phantoms.

The X-PET™ and LabPET-8™ small animal PET scanners commercialized by GE Healthcare (Waukesha, WI) were used in this work. The X-PET™ is a Bi_4_Ge_3_O_12_ (BGO)-based small animal PET scanner, having an axial FOV of 11.6 cm [22, 23]. The system incorporates various technological innovations to improve system performance [7]. For instance, the photomultiplier-quadrant-sharing method is used to maximize the number of crystals per photomultiplier whereas a high-yield pileup event recovery electronic processing technology is exploited to improve the count-rate performance [24]. The detector blocks are circularized by slightly grinding each block on the photomultiplier side into a pentagon shape. On the other hand, the LabPET-8™ is an avalanche photodiode (APD)-based digital PET scanner having a 7.5 cm axial FOV, designed with quasi-individual crystal readout along with parallel digital architecture to achieve high-performance [11]. Scintillation crystals composed of Lu_0.4_Gd_1.6_SiO_5_ (LGSO) and Lu_1.9_Y_0.1_SiO_5_ (LYSO) are optically coupled one after the other, forming phoswich pairs of detectors that are read out by a single APD. Four phoswich detectors are enclosed in a hermetic container made of kovar (an iron-nickel-cobalt alloy with a density of 8.359 g/cm^3^) having external dimensions of 10.3 × 4.7 × 18 mm. The end of axial FOV shielding is made of tungsten (19.3 g/cm^3^ density, 15.75 mm thickness and 131 mm internal diameter) to minimize the detection of out-of-FOV activity [11]. The most relevant design features of both scanners are summarized in Table 1.

**Table 1:** Summary of technical specifications of the X-PET™ and LabPET-8™ preclinical PET scanners

Type	X-PET™	LabPET-8™
Scintillator	BGO	Phoswich pair of LYSO/LGSO
Crystal dimension	2.32 × 2.32 × 9.4 mm^3^	2.0 × 2.0 × 14 mm^3^
No. of detector rings	48	32
Crystals per ring	240	192
Total no. of crystals	11,520	6,144
Detector ring inner diameter (mm)	165	162
Transaxial field-of-view (mm)	100	100
Axial field-of-view (mm)	116	75
Image pixel size (mm)	0.4	0.5
Slice thickness (mm)	0.4	0.25/0.5

For the X-PET™, pentagonal detector blocks were modeled using trapezoidal volumes. An energy resolution of 25% for 511 keV photons was applied as blurring kernel for energies deposited within the crystals [25]. The LET was set to 250 keV whereas the higher energy threshold was set to 750 keV in all simulations. These energy thresholds were chosen to mimic default energy threshold settings on the actual X-PET™ scanner.

For the LabPET-8™, the GATE materials database was modified to add LYSO, LGSO and kovar. For both LYSO and LGSO, an energy resolution of 25% and an average timing resolution of 9 ns were set [26]. The coincidence window was set to 20 ns. The LET was set to 250 keV whereas the higher energy threshold was set to 650 keV. These energy thresholds and coincidence window settings are similar to the settings on the actual LabPET-8™ scanner.

Back-to-back 511 keV annihilation photons were generated to decrease computational time. The animal positioning bed was not modeled for both scanners. The GATE output information was recorded in ASCII format and then rebinned into sinograms using single-slice rebinning (SSRB) [27].

The validation parameters are calculated in according to NEMA-04 procedures. The SF and NECR were calculated for mouse and rat phantoms using radioactive line sources radially offset by 10 and 17.5 mm, respectively. In brief, for each total events sinogram, all pixels located farther than 8 mm from the edge of the phantom were set to zero. The profile of each projection angle was shifted so that the maximum valued pixels were aligned with the central pixel of the sinograms. A sum projection was obtained by adding up all angular projections in each slice. A linear interpolation between the left and right edges of the 14 mm central band was used to differentiate the trues from other counts.

The total event rate *R*_tot,*i*_, for each acquisition of each slice *i* is computed as:

1 $$ {R_{{{\text{tot}},i}}} = \frac{{{C_{{{\text{tot}},i}}}}}{{{T_{\text{acq}}}}} $$

where *T*_acq_ is the acquisition time. The system total event rate, *R*_tot_, is computed as the sum of *R*_tot*,i*_ over all slices *i*.

The SF for each acquisition was computed using the following formula:

2 $$ {\text{SF}} = \frac{{{R_{\text{scatter}}}}}{{{R_{\text{true}}} + {R_{\text{scatter}}}}} $$

The NECR, *R*_nec_, for each acquisition was computed as:

3 $$ {R_{{nec}}} = \frac{{R_{{true}}^2}}{{{R_{{total}}}}} $$

The peak absolute sensitivity was measured using the ^22^Na point source as described in NEMA-04 procedures and is given by:

4 $$ {S_{\text{a}}} = \frac{{{S_{\text{i}}}}}{{0.906}} \times 100 $$

where 0.906 is the branching ratio of ^22^Na and *S*_i_ is the sensitivity (in counts/s/Bq). The data were processed using programs developed in-house implemented in MATLAB 7.4 (Mathworks, Natick, MA).

The X-PET™ simulation model was validated against experimental results [7] whereas the LabPET-8™ simulation model was validated against experimental results performed by our group and those reported by Bergeron *et al.* [28] in terms of sensitivity, NECR and SF for mouse and rat-sized phantoms. The relative difference is calculated as the percentage difference relative to the mean.

### Design and Fabrication of the Cone-Shaped Phantom

**Fig. 1:**
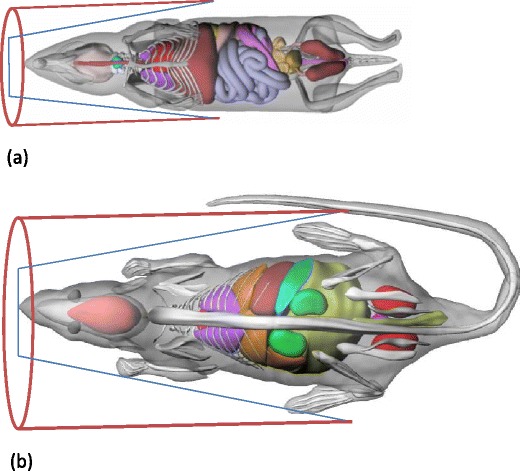
Illustration of rodents’ shape in the axial direction using NURBS-based preclinical anatomical models corresponding to **a** mouse and **b** rat.

Typical shapes of mouse and rat bodies represented by non-uniform rational B-spline (NURBS)-based mouse [29] and rat [30] models are shown in Fig. 1. It can be seen that the upper part of rodent bodies bears a resemblance to a tapered than a cylindrical shape. The parameters considered for phantom design are material composition, cross-sectional dimensions and taper angle (*θ*). The fabrication material chosen is high density polyethylene (density of 0.96 ± 0.1 g/cm^3^) as prescribed in the NEMA-NU04 standards. The maximum diameter of the phantom was derived from the transaxial FOV of various small animal scanners [6, 7, 9, 11, 31]. Based on this assessment, the maximum diameter of the phantom was set to 70 mm by considering 70% of the average maximum diameter of the transaxial FOV. The minimum diameter of the phantom was set to 20 mm because of limitations of the milling machine used. The length of the phantom depends on the taper angle. The length *L* and the taper angle *θ* are related by the following equation:

5 $$ L = \left( {{\text{Maximum}}\;{\text{diameter}} - {\text{minimum}}\;{\text{diameter}}} \right)/\left( {2 \times \tan \theta } \right) $$

**Fig. 2:**
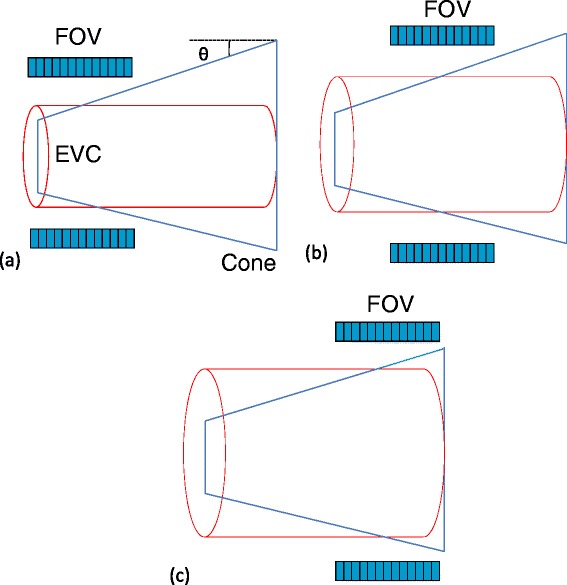
Illustration of the cone-shape phantom and its equivalent volume cylindrical (*EVC*) phantom for the various axial fields-of-view: **a** minimum (FOV_mouse_), **b** middle (FOV_rat_) and **c** maximum (FOV_rabbit_) diameter region in the scanner’s axial FOV.

The corresponding phantom length is 28.6, 20.4, 15.8 and 12.9 cm for taper angles of 5°, 7°, 9° and 11°. As *θ* increases, the phantom length decreases, thus increasing the range of the cross-sectional volume covering one axial FOV, making it less representative of an equivalent volume cylindrical (EVC) phantom. However, for small *θ*, the phantom becomes long enough for the corresponding small animal PET axial FOV. The EVC phantom is defined as a phantom having a uniform cylindrical shape and diameter equal to the diameter of the cone-shaped phantom in the middle of the axial FOV of the scanner (Fig. 2). When *θ* = 0°, the cone-shaped phantom corresponds to the EVC phantom having diameter of minimum or maximum side of the cone-shaped phantom. Therefore, the optimization of the taper angle is crucial for the design of the cone-shaped phantom. Different cone-shaped phantoms with minimum diameter of 2 cm, maximum diameter of 7 cm and taper angles of 5°, 7°, 9° and 11° were simulated using the Monte Carlo model of the X-PET™ scanner described in “Modeling and Validation of Small Animal PET Scanners”. Line sources of different length were placed in the center of the phantom. Simulations were performed for different taper angles of 5°, 7°, 9° and 11° and its corresponding EVC phantom for central region of phantom. The corresponding diameter of the EVC phantom for taper angles of 5°, 7°, 9° and 11° were 5.5, 5, 4.5 and 4 cm, respectively. The taper angle was optimized by analyzing the simulated data of the X-PET™ scanner in terms of SF, NECR and true count rate using the NEMA-NU04 standards as described in “Modeling and Validation of Small Animal PET Scanners”.

The phantom was fabricated in-house considering the optimized design considerations discussed above including material, cross-sectional dimensions and taper angle. To study the effects of radial offsets, four holes (4 mm diameter) were drilled parallel to the long axis at the center and at radial offset positions of 10, 15 and 20 mm. Solid line fillings of the same material used for the phantom were also fabricated. These fillings were used to fill up the remaining holes when one particular hole is filled with a radioactive line source during acquisition.

The different FOVs of the cone-shaped phantom corresponding to minimum, middle and maximum diameter region in the scanner’s axial FOV will be referred to as FOV_mouse_, FOV_rat_ and FOV_rabbit_, respectively. The phantom dimensions of each FOV for the X-PET™ and LabPET-8™ scanners are summarized in Table 2. The cone-shaped phantom can be dismantled into three equal parts, thus allowing various combinations to match the axial FOV of different small animal PET scanners. Depending on the scanner’s axial FOV, the cone-shaped phantom can be exploited to simulate various sizes of mice, rats and small rabbits used in small animal PET imaging. For instance, in the case of the LabPET-8™, FOV_mouse_ (diameter from 2 to 4.5 cm), FOV_rat_ (diameter from 3 to 6 cm) and FOV_rabbit_ (diameter from 4.5 to 7 cm), can be used to simulate the various sizes of these species to assess variations in SF and NECR.

**Table 2:** Dimensions of different FOVs of the cone-shaped and EVC phantoms for the X-PET™ and LabPET-8™ scanners

Scanner	FOV	Cone-shaped phantom			EVC phantom	
		Min. diameter (cm)	Max. diameter (cm)	Length (cm)	Diameter (cm)	Length (cm)
X-PET™	FOV_mouse_	2	6	11.6	4	11.6
	FOV_rat_	2.5	6.5	11.6	4.5	11.6
	FOV_rabbit_	3	7	11.6	5.0	11.6
LabPET-8™	FOV_mouse_	2	4.5	7.5	3.2	7.5
	FOV_rat_	3	6	7.5	4.5	7.5
	FOV_rabbit_	4.5	7	7.5	5.8	7.5

### Studies using the Cone-Shaped Phantom

The optimized cone-shaped phantom was further used to assess the magnitude of scatter and NECR as function of varying phantom size, LET and line source position for both small animal PET scanners using both Monte Carlo simulation and experimental studies.

#### Simulation Studies

 PET data were simulated and analyzed for a line source at the center and at radial offsets of 10, 15 and 20 mm for LETs of 250, 350 and 425 keV, whereas the higher energy threshold was kept constant (650 keV for LabPET-8™ and 750 keV for the X-PET™). Back-to-back 511 keV annihilation photons were simulated for one line source at a time (10^9^ events). All simulations were performed at a low radioactivity regime as defined in the NEMA-04 standards for SF calculation. Data were collected at three different axial FOVs from one end of the phantom to the other in three successive axial steps, namely FOV_mouse_, FOV_rat_ and FOV_rabbit_. An EVC of the cone-shaped phantom at three different axial FOVs was also simulated.

#### Experimental Studies

 Simulation studies using the cone-shaped phantom were experimentally validated for the LabPET-8™ scanner. The line source was filled with 2 MBq of ^18^F and inserted in the central hole of the phantom while the other holes were closed using the fillings. The phantom was placed on the scanner bed using Styrofoam support at the smaller end of the phantom. Adhesive tape was also used to secure the phantom on the scanner bed. The data were acquired for three FOVs, namely FOV_mouse_, FOV_rat_ and FOV_rabbit_ one after the other and were corrected for radioactive decay. This was repeated for other holes and always a new line source filled with 2 MBq of ^18^F was used. Each acquisition lasted 5 min. PET studies were acquired using a default energy window of 250–650 keV and 10/15/20 ns time window for LYSO-LYSO/LYSO-LGSO/LGSO-LGSO coincidences in list-mode format which were binned into 3D sinograms. These 3D sinograms were further rebinned to 2D sinograms using SSRB. These 2D sinograms were used for further analysis adhering to NEMA-NU04 standards. The relative difference (in percent) between simulated and experimental results was also calculated.

## Results

### Cone-Shaped Phantom

**Fig. 3:**
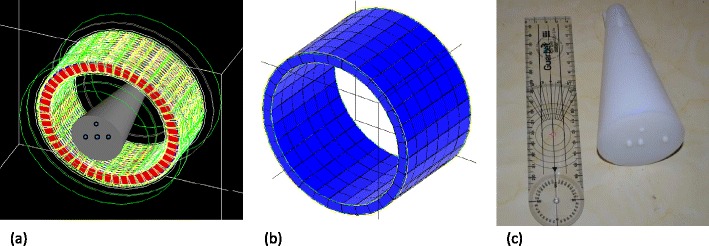
Simulation models using GATE of **a** the LabPET-8™ scanner and **b** the X-PET™ scanner. **c** Photograph of the fabricated cone-shaped physical phantom.

**Fig. 4:**
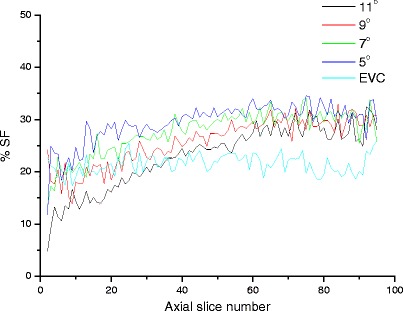
Plots of SF (in percent) versus axial slices for different taper angles of the cone-shaped phantom and equivalent cylindrical volume (*EVC*) phantom.

Figure 3a-b illustrates the modelled LabPET-8™ and X-PET™ using GATE toolkit. The developed physical cone-shaped phantom is shown in Fig. 3c. The optimal minimum and maximum diameters of the cone-shaped phantom are 2 and 7 cm, respectively. The total and true events rate, NECR, SF and total phantom length for different taper angles of the cone-shaped phantom are summarized in Table 3. Out of four simulated taper angles, an angle of 11° produced the highest total, true and NECR events rate whereas it resulted in the lowest SF. Figure 4 presents the SF profile for slice numbers covering the axial FOV of the X-PET™ scanner. It can be observed that the SF profiles for 9° and 11° are closer to the SF profile of the EVC phantom. Since a maximum taper angle of 10° was not possible for the milling machine used to fabricate the phantom due to its limitations, a taper angle of 9° was chosen.

**Table 3:** Summary of total and true events rate, NECR and SF for different taper angles of the cone-shaped phantom for the X-PET™ scanner

Taper angle (θ)	Total events rate	True events rate	NECR	SF	Phantom length (cm)
11°	31,594	24,024	18,329	23.96	12.9
9°	31,194	22,957	16,933	26.41	15.8
7°	30,734	21,932	15,677	28.64	20.4
5°	30,388	21,152	14,741	30.39	28.6

### Experimental and Simulation Studies

The validation results of the simulated X-PET™ and LabPET-8™ models are summarized in Table 4. It can be seen that there is good agreement between simulated and measured values for all considered parameters with a relative difference varying between 4.8% and 10.8%. This gives confidence to use the simulated models for prediction of performance parameters under various conditions. For Monte Carlo simulation model validation, the line source position was set according to NEMA-NU04 standards, that is, 10 mm for the mouse phantom and 17.5 mm for the rat phantom, radially offset from the center.

**Table 4:** Comparison between simulated and measured performance parameters of the X-PET™ and LabPET-8™ small animal PET scanners

Parameter	X-PET™			LabPET-8™		
	Simulated	Measured^a^	Relative difference (%)	Simulated	Measured^b^	Relative difference (%)
SF (%) mouse phantom	7.2	7.9	9.2	18.10	19	4.8
SF (%) rat phantom	19.1	21	9.4	28.18	31	9.5
Peak NECR (kcps) mouse phantom	114	106	7.2	204	183	10.8
Absolute Sensitivity (%)	6.3	5.9	6.5	1.46	1.33	9.3

^a^Data taken from Ref. [7]

^b^Data taken from Ref. [28]

**Fig. 5:**
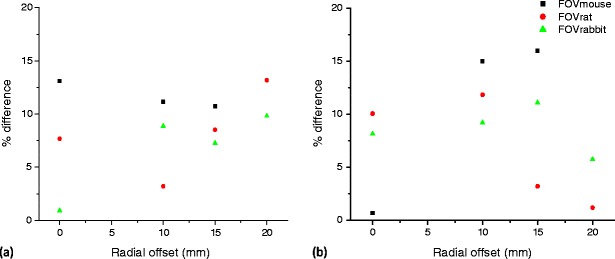
Relative difference between simulated and experimental results for the LabPET-8™ scanner in terms of SF (**a**) and NECR (**b**).

Experimental studies performed using the cone-shaped phantom were acquired on the LabPET-8™ scanner. Figure 5 shows the percent relative difference between simulated and experimental results for the LabPET-8™ scanner in terms of NECR and SF as function of line source radial position for the three considered FOVs (FOV_mouse_, FOV_rat_ and FOV_rabbit_). The resulting relative differences using an energy window of 250–650 keV vary mostly between 0.7% and 10% and overall remain below 16%.

**Fig. 6:**
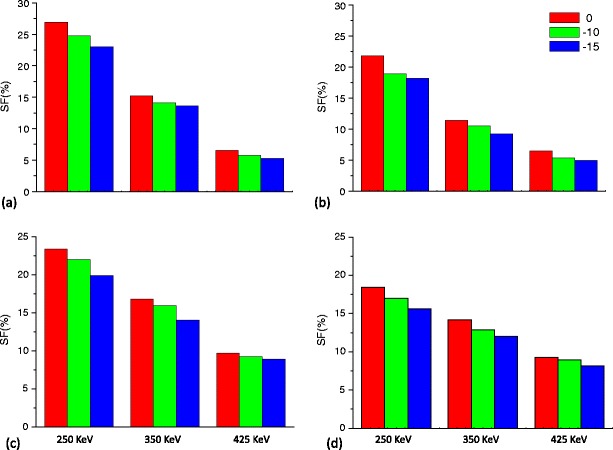
Variation of SF (in percent) as a function of radial offsets using a LET of 250, 350 and 425 keV for axial FOV_mouse_. **a** The LabPET-8™ cone-shaped phantom, **b** LabPET-8™ EVC phantom, **c** X-PET™ cone-shaped phantom, **d** X-PET™ EVC phantom.

**Fig. 7:**
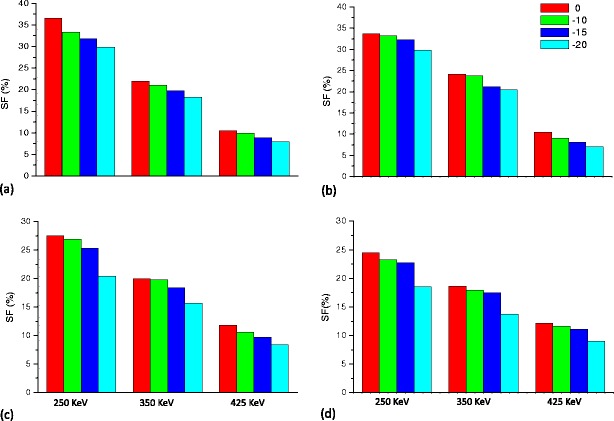
Variation of SF (in percent) as a function of radial offsets using a LET of 250, 350 and 425 keV for axial FOV_rabbit_. **a** The LabPET-8™ cone-shaped phantom, **b** LabPET-8™ EVC phantom, **c** X-PET™ cone-shaped phantom, **d** X-PET™ EVC phantom.

The SF for FOV_mouse_ corresponding to the cone-shaped and EVC phantoms for both the X-PET™ and LabPET-8™ scanners using a LET of 250, 350 and 425 keV is shown in Fig. 6. The SF estimates are shown for a line source located at the center and at 10 and 15 mm radial offset. Similar results are shown in Fig. 7 for FOV_rabbit_. In the former instances, a line source with radial offset of 20 mm was not considered since the minimum diameter for FOV_mouse_ is 20 mm. Using the cone-shaped phantom, the SF for FOV_rat_ varies as a function of the radial offset of the line source. The range of this variation is from 26.3% to 18.2%, 18.6 to 13.1% and 10.1 to 7.6% for the X-PET™, whereas it was from 34.4% to 26.9%, 19.1 to 17.0% and 9.1 to 7.3% for the LabPET-8™, for LETs of 250, 350 and 425 keV, respectively. For the EVC phantom, the SF varied from 21.8% to 15.5%, 17.0 to 11.3% and 10.7 to 7.6% for the X-PET™, whereas it varied from 29.8% to 23.8%, 18.6 to 14.4% and 9.1 to 7.0% for the LabPET-8™, for LETs of 250, 350 and 425 keV, respectively.

**Fig. 8:**
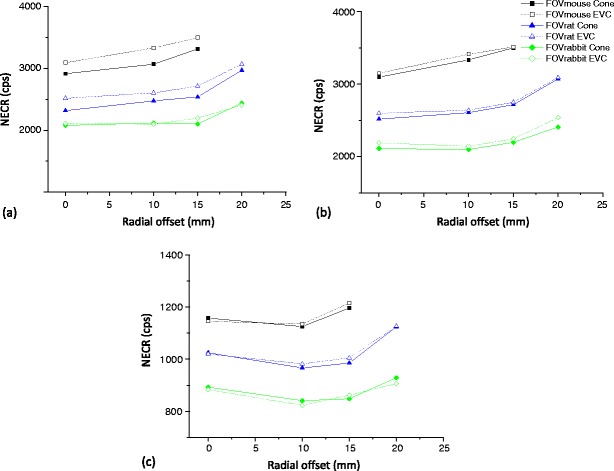
**a** Trends of the LabPET-8™ NECR as function of radial offsets for the cone-shaped and EVC phantoms using different LETs: **a** 250, **b** 350 and **c** 425 keV.

**Fig. 9:**
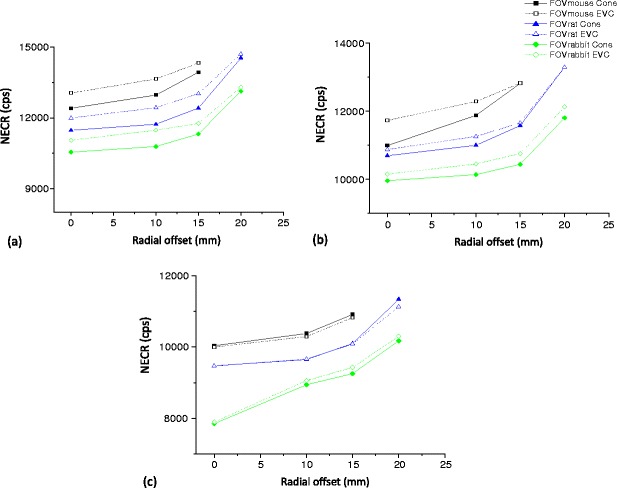
Trends of the X-PET™ NECR as function of radial offsets for the cone-shaped and EVC phantoms using different LETs: **a** 250, **b** 350 and **c** 425 keV.

Figure 8 shows the NECR for the LabPET-8™ scanner for both the cone-shaped and EVC phantoms for three axial FOVs versus the line source radial offset when using LETs of 250, 350 and 425 keV. The same parameter is plotted for the X-PET™ scanner in Fig. 9. Table 5 highlights the high correlation (*R* ^2^) between the SF and NECR estimates for the cone-shaped phantom and the EVC phantom for the three axial FOVs for both scanners.

**Table 5:** Correlation coefficient (*R* ^2^) for NECR and SF between the cone-shaped phantom and EVC phantom for both the X-PET™ and LabPET-8™ scanners

Scanner	LET	FOV_mouse_		FOV_rat_		FOV_rabbit_	
		SF	NECR	SF	NECR	SF	NECR
X-PET™	250	0.990	0.999	0.998	0.996	0.992	0.995
	350	0.950	0.999	0.996	0.999	0.982	0.998
	425	0.965	0.999	0.895	0.996	0.951	0.960
LabPET-8™	250	0.963	0.973	0.999	0.995	0.885	0.946
	350	0.958	0.988	0.932	0.997	0.954	0.993
	425	0.990	0.999	0.983	0.990	0.988	0.923

As expected, the SF for both scanners decreases as the radial offset increases, LET increases and object size decreases. However, in all cases the SF of the LabPET-8™ is higher than the X-PET™ scanner. The NECR for both scanners increases as the radial offset increases and object size decreases. The NECR reached a maximum for the LabPET-8™ at a LET of 350 keV whereas it reached the maximum value at a LET of 250 keV for the X-PET™. However, in all cases the NECR for the X-PET™ is higher compared with the LabPET-8™ scanner.

## Discussion

The SF and NECR are important parameters for optimization of acquisition protocol settings such as timing and energy windows and comparing the performance of different small animal PET scanners. In addition, the SF is useful for the assessment of the relevance and level of complexity of scatter correction required for small animal PET studies. These parameters are usually measured using various discrete uniform phantoms of different size [14–16]. Reproducibility of measurements and shape/size of the phantoms are some limitations of this approach. The analysis of body shape of various voxel-based, NURBS-based models and actual laboratory animals revealed that the shape of the upper body of rodents resembles more to tapered shape than cylindrical shape (Fig. 1). It should also be emphasized that there is considerable variation in body size within particular rodents’ species, especially rats and small rabbits. This motivated the design and fabrication of a single cone-shaped phantom suitable for assessing object size-dependent SF and NECR of small animal PET systems. It should be noted that the purpose is not to replace or suggest an alternative to well established NEMA standards [17]. The phantom design was optimized using Monte Carlo simulation studies of the X-PET™ scanner using the GATE toolkit. The validation of simulation models of both scanners against experimental measurements proved that the models are capable of predicting the response of actual systems with acceptable accuracy. Overall, there was good agreement between simulated and experimental results for both scanners, with a relative error varying between 4.8% and 10.8%. The methodology followed for the design and optimization of the cone-shaped phantom is an extension of the work described by Wilson *et al.* [18] in the context of clinical PET to small animal imaging. The phantom described in the reference above is a fillable tapered design suitable for clinical imaging, whereas we opted for a solid tapered phantom dedicated for small animal PET imaging having multiple holes to insert line sources at different radial offset positions.

The phantom design parameters that had to be optimized are minimum and maximum diameters, length of the cone and taper angle. Although the determined optimal taper angle is 11°, a taper angle of 9° was used for fabricating the phantom owing to the limitations of the milling machine. Using the cone-shaped phantom, the difference between simulated and experimental SF and NECR results for the LabPET-8™ scanner is below 16%. It should be noted that we have not considered the positron range, animal bed, the intrinsic radioactivity emanating from LGSO and LYSO crystals in the simulation model. Inclusion of positron range in the simulation model can affect the scatter fraction estimates, especially for smaller animal sizes. However, this may not have a significant effect for the low energy positrons of ^18^F [16]. On the other hand, Rechka *et al.* [26] have shown that the Compton scattering probability due to scanner bed is small (2.8%). Experimental studies using the cone-shaped phantom could not be performed on the X-PET™ scanner since it was upgraded to LabPET-8™ and as such is no longer available for further investigation.

The SF decreases as the radial offset increases from center to radial offset of 20 mm, LET increases from 250 to 425 keV and, object size decreases from small rabbit body size to mouse body size. The NECR for both scanners increases as the radial offset increases and object size decreases. The maximum NECR is achieved at a LET of 350 keV for the LabPET-8™ and 250 keV for the X-PET™. This reflects the effect of radial position, object size and LET on SF and NECR, which in turn affects image quality and quantitative accuracy of small animal PET imaging. Similar behaviour has been reported elsewhere [14]. The SF for the LabPET-8™ is higher than for the X-PET™ when using the same settings. This can be attributed to the detector housing and FOV shielding of the LabPET™ scanner which increases the signal detection efficiency by 23% [26], the higher sensitivity to out-of-FOV activity for the LabPET-8™ owing to the shorter axial FOV, inter-crystal scattering which is higher for LYSO/LGSO compared with BGO [32] and the inherent scintillation crystal material characteristics [33]. Moreover, the difference between the SF of the LabPET-8™ and X-PET™ scanners decreases as the LET increases. Overall, the maximum SF observed using the cone-shaped phantom for mouse, rat and small rabbit animals using a LED of 250 keV is 23.3%, 34.4% and 36.5%, respectively. This includes the scatter from objects under study, scanner gantry and surrounding environment. This suggests that scatter correction is important for the accurate quantification in small animal PET imaging [34].

High correlation coefficients for SF and NECR were observed between the cone-shaped phantom and the EVC phantom for different axial FOVs. This seems to indicate that the cone-shaped phantom can be equally used as the uniform EVC phantom to simulate different small animal body sizes. Similar conclusions were drawn by Wilson *et al.* [18] in the context of clinical PET imaging.

An interesting aspect of the cone-shaped phantom is that it allows the assessment of the effect of three parameters, namely radial offset, energy threshold and object size in a single acquisition. The cone-shaped phantom can be used for evaluation of the effects of radial offsets when imaging several mice simultaneously [19]. Regions corresponding to FOV_mouse_, FOV_rat_ and FOV_rabbit_ in the cone-shaped phantom can be used to assess the SF and NECR for various sized animals including mice, rats and small rabbits. Since the cone-shaped phantom can be dismantled into three equal parts, it can be used in various combinations to cope with the axial FOV of different small animal PET scanners.

## Conclusions

A cone-shaped phantom was designed and fabricated for assessment of object size-dependent SF and NECR for small animal PET scanners. The characteristics of the LabPET-8™ and X-PET™ small animal PET scanners were studied using this phantom in terms of SF and count rate analysis. A single cone-shaped phantom enables the assessment of the effect of three factors, namely radial offset, energy threshold and object size on the SF and NECR for various sizes of mice, rats and small rabbits. This makes the cone-shaped phantom suitable for evaluation of object size-dependent SF and NECR for small animal PET imaging.
